# On-Site Bioaerosol Sampling and Airborne Microorganism Detection Technologies

**DOI:** 10.3390/bios14030122

**Published:** 2024-02-24

**Authors:** Afagh Rastmanesh, Jayanta S. Boruah, Min-Seok Lee, Seungkyung Park

**Affiliations:** Complex Fluids Laboratory, School of Mechanical Engineering, Korea University of Technology and Education, Cheonan 31253, Chungnam, Republic of Korea

**Keywords:** bioaerosol, infectious disease, sampling methods, detection, pathogens

## Abstract

Bioaerosols are small airborne particles composed of microbiological fragments, including bacteria, viruses, fungi, pollens, and/or by-products of cells, which may be viable or non-viable wherever applicable. Exposure to these agents can cause a variety of health issues, such as allergic and infectious diseases, neurological disorders, and cancer. Therefore, detecting and identifying bioaerosols is crucial, and bioaerosol sampling is a key step in any bioaerosol investigation. This review provides an overview of the current bioaerosol sampling methods, both passive and active, as well as their applications and limitations for rapid on-site monitoring. The challenges and trends for detecting airborne microorganisms using molecular and immunological methods are also discussed, along with a summary and outlook for the development of prompt monitoring technologies.

## 1. Introduction

Bioaerosols are defined as small airborne microbiological particles including pathogenic or non-pathogenic bacteria, viruses, fungi, spores, archaea, or other fragments [1]. They also consist of pollens, other organic matter, toxins, and their by-products, such as peptidoglycans, allergens with high molecular weight, endotoxins, and mycotoxins, and they can be either viable or non-viable in nature wherever applicable [2]. They are dispersed in the ambient air. They range from micro-sized viral particles to 1 mm pollen grains. Moreover, they are present in a mixture of droplets, salts, dust, etc. So, it is necessary to efficiently separate and collect the target bioaerosols in the environment prior to bioanalysis [3,4].

**Scheme 1:**
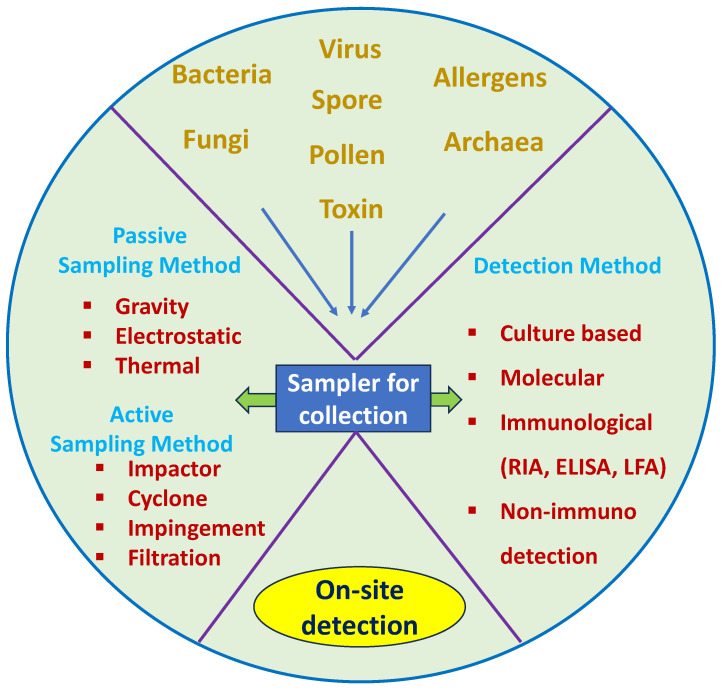
The process of bioaerosol sample collection with different samplers and available methods of detection.

The detection and identification of bioaerosols has assumed great importance in the last few decades because of their effects on human health [1]. Recently, the increasing threat of new or re-emerging infectious diseases such as COVID-19 brought about an urgent need for an efficient method for monitoring bioaerosols to minimize the impact on public health. In fact, the accurate, timely, and highly sensitive surveillance of microbial aerosols in indoor and outdoor air is the primary step to prevent and control such diseases [5]. Although various technologies and protocols for the detection or identification of microorganisms in laboratory-based settings exist, rapid and on-site bioaerosol monitoring methods are severely limited mainly due to their low concentration and diversity in indoor or outdoor environments. There are many analytical techniques used for such bioaerosol monitoring, and their advantages and disadvantages have been described systematically by Santarpia and coworkers [6]. Thus, bioaerosol monitoring essentially requires effective sampling methods followed by appropriate detection techniques for the collected samples. However, the lack of standard and field-applicable strategies hinders on-site monitoring. This review focuses on the available bioaerosol sampling and detection technologies and discusses their potential for on-site monitoring applications. Basically, it covers the importance of bioaerosols with respect to human health; details the available bioaerosol samplers, the types of samplers, and their architectures, advantages, and limitations; and discusses the sampling process considering all types of bioaerosol members, as well as different types of detection methods. In each section, we reference previous works to show their efficacy and improvement trends step by step. Further, the limits of detection of sensing/detection platforms are described and compared to propose suggestions regarding what bioaerosol researchers could explore further. The selection of a method for sampling and detection is dependent on the properties of the target bioaerosols, and it is realized through the discussion in the paper. Finally, we summarize the whole discussion by providing comparative statements and pointing out the things to consider for an effective bioaerosol sampler and detection platform. A schematic representing the topics discussed throughout this paper is shown in Scheme 1.

## 2. Bioaerosol Sampling

Bioaerosols are commonly recognized by their physical properties (such as shape and size), chemical composition (organic elemental ratio), nucleic acids (RNA, DNA), energy-carrying compounds (ADP), and other structural compounds (cellulose) [7]. In humans, exposure to these microbiological agents can cause a wide range of infectious diseases, respiratory diseases, acute toxic effects, neurological effects, and cancer. Therefore, the presence of bioaerosols in different environments and their impact on human health must be sampled and analyzed [8]. 

**Figure 1:**
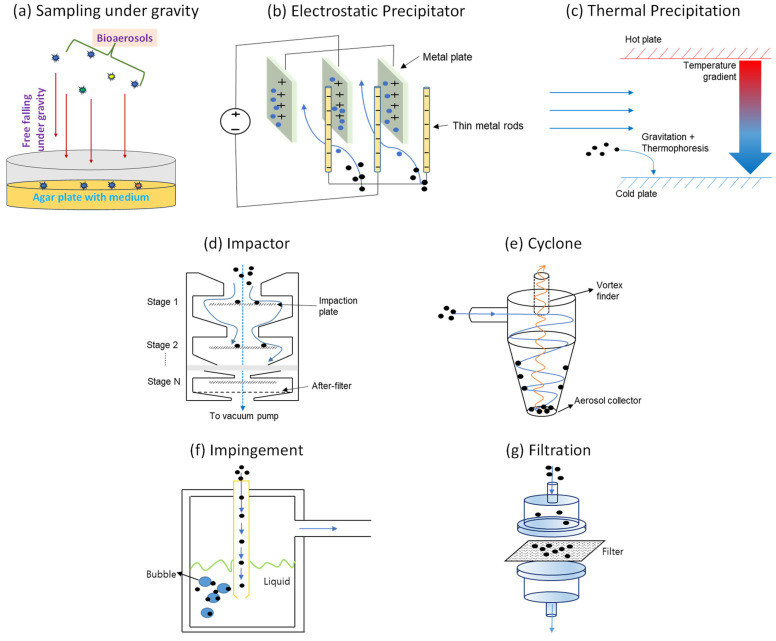
Types of passive (**a**–**c**) and active (**d**–**g**) sampling methods.

Many traditional and modern techniques are used to investigate and detect bioaerosol samples’ properties. A wide variety of bioaerosol samplers are commercially available, and there are many more currently in development. However, most samplers broadly fall into two major categories: passive sampling and active sampling. Figure 1 shows schematics of the sampling methods discussed in this section, and Table 1 summarizes the pros and cons of each method.

### 2.1. Passive Sampling

Passive sampling is a portable, compact, and inexpensive method of bioaerosol sampling that operates based on gravity, electrostatic force, their combination, and turbulent dispersion [9]. It relies on the free flow of analyte molecules from the sampled medium to a collecting medium, driven by the chemical potential difference between the two media. Compared to active sampling, passive sampling methods offer many advantages, including their simplicity, low cost, and the lack of a need for expensive pumps, complicated equipment, or unattended operation. Passive sampling also has the added benefit of being noiseless, and it can be used in hazardous environments, producing accurate results [10].

Traditionally, passive sampling has been used to determine culturable bioaerosols by using settling agar plates. However, this method has the major drawback of the air volume being unspecified, making it a non-quantitative technique, or more simply, a qualitative technique. It only determines sedimentation rate, not the exact concentration. The diffusion-based particle flux is relatively poor and varies with respect to particle size. Nonetheless, new technical developments in passive sampling suggest that it can be a complementary technique to active sampling [11]. The involvement of a filtration process is common in both passive and active samplers. This is because, in order to collect samples, both quantitative and qualitative techniques require filter paper to be put in the direction of the air flow or in the sedimented platform. Also, to remove other contaminants from the bioaerosol, filter paper or general filters are widely used. Passive samplers are generally categorized based on their collecting mechanism, such as sampling under gravity, electrostatic attraction, and thermal precipitation [9]. The following sections will provide detailed discussions of these mechanisms.

#### 2.1.1. Passive Sampling under Gravity

As mentioned above, the simplest method for bioaerosol sample collection is to collect samples under the influence of gravitational force. For this, filter paper or any such depositing material will be helpful. Some gravity-based samplers are agar settle plates, settling filters, the Einstein–Lioy Sampler, dust fall collectors or Petri dishes, Durham-type passive spore traps, remote airborne microbial passive (RAMP) samplers, the personal aeroallergen sampler (PAAS), etc. [9]. Regarding agar settle plates, agar media plates (100 mm) are kept in the sampling environment for sample collection for up to 4 h. Moreover, different filters can be applied for settling down as required depending on the sample environment and position. In the case of Einstein–Lioy sampler, four filters are connected simultaneously in a filter holder to avoid the wind effect. Sometimes, a box or sterile Petri dish known as a dust fall collector is used for sample collection. Durham-type traps help in the protection of slides kept outdoors for long-time sampling. For RAMP samplers, gel-coated square Petri dishes are employed for high-altitude sample collection. The PAAS is a personal sampler that is worn around the person’s breathing zone. 

#### 2.1.2. Passive Sampling through Electrostatic Attraction

Electrostatic bioaerosol samplers use electrostatic attraction to capture charged particles by charging the aerosol. Recent studies have shown that electrostatic methods are some of the most suitable methods for collecting microorganisms, and interest in this sampling method is growing. Compared to other collection methods, electrostatic sampling has several advantages, such as lower impaction stress, lower pressure drops, and lower power consumption. One of the most attractive features of electrostatic samplers is their lower particle deposition compared to other inertia-based methods, which suggests that electrostatic collectors cause less damage to microorganisms. Moreover, electrostatic samplers are compatible with multiple collection media surfaces, such as agar, liquid, and solid media [12].

The electrostatic method was first adapted by Berry and his co-authors for collecting bacteria (*Serratia marcescens*) from air. They modified the funnel devices for the electrostatic method by adding more forces via the electrostatic principle [13]. In the 1960s, Morrow et al. described a point-to-plane electrostatic precipitator. They used an electron microscope to develop a particle size sample instrument [14]. Then, in 1985, Liu and his co-workers studied electrostatic effects in relation to aerosol sampling and filtration. They also studied the nature of particle deposition in various types of sampling tubes. The results of their study concluded that tygon-shaped tubing has better efficiency and also has relatively small electrostatic effects [15].

The interest in applying electrostatic samplers for bioaerosol sampling decreased from the 1980s to the late 1990s due to the rapid advancement of other sampling methods. However, the interest in low-power sampling techniques and concerns about the adverse effects of other inertia-based processes led to a resurgence in the interest in electrostatic samplers. In 1999, Mainelis and colleagues developed a stand-alone electrostatic sampler for collecting culturable microorganisms in both laboratory and field experiments using the modified Electrostatic Aerosol Sampler (Model 3100, TSI Inc., St. Paul, MN, USA) [16]. In the same year, German researchers designed and constructed a new electrostatic precipitator for off-line particle analysis, optimizing the collection efficiency and deposition pattern of gasborne particles using numerical methods and experiments [17]. Gast and colleagues developed an electrostatic sampling device for the detection of airborne *Salmonella enteritidis* in the environment of experimentally infected laying hens, achieving significantly better *Salmonella enteritidis* detection [18]. In 2008, Madsen and Sharma developed a new high-volume electrostatic field sampler for sampling high amounts of bioaerosols, achieving better results through the use of a strong electrostatic field [19]. 

In large-scale population studies, active airborne dust sampling can be limited by logistic and financial constraints [17]. To address this issue, a simple and low-cost electrostatic dust fall collector (EDC) was developed and evaluated in 2008 for indoor air endotoxin exposure assessment, a key issue in asthma and allergy. The EDC consisted of a folder that was 42 cm by 29.6 cm in size with four electrostatic cloths exposed to the air. Active airborne dust sampling was also performed in parallel, and the results showed that measuring endotoxin levels with the EDC is a valid measure of average airborne endotoxin exposure [20]. In the late 2000s, electrostatic precipitation for collecting bioaerosols gained great attention due to its higher biological collection efficiency compared to the Andersen-type impactor BioStage, with 5–9 times greater efficiency being reported. Importantly, the electrostatic field did not cause any damage to the culturability of the collected airborne microorganisms.

In the 21st century, new and different types of electro samplers have been developed and investigated. One such device is the integrated microfluidic electrostatic sampler (IMES), which was developed and evaluated by Ma and his co-workers [21]. This sampler consists of a unipolar charging chamber, a half-cylinder precipitation electrode, and a collection chip for automated air sample delivery. The developed air sampler achieved a better collection efficiency of about 40% at a charging voltage of −1.8 kV and collecting voltage of −7 kV. Another electrostatic sampler with a high flow rate of 100 L/min was developed by Roux and his co-workers [22]. This sampler was the first electrostatic sampler developed with a high flow rate and was found to be an efficient single-static electrostatic sampler.

Han and his co-workers recently developed a new type of electrostatic precipitator with a super hydrophobic surface that collects particles into 10 to 40 µL of water droplets, allowing for the achievement of very high concentration rates, and they analyzed the performance of this sampler with three different fungal spores: *Cladosporium cladosporioides*, *Penicillium melinii*, and *Aspergillus versicolor* [23]. In another study, a field-deployable version of the electrostatic precipitator with superhydrophobic surface (FDEPSS) was developed to test two bacterial species, *Bacillus atropheus* and *Pseudomonas fluorescens* bacteria, and one fungal spore, *Penicillium chrysogenum*. The collection efficiency was found to be ∼70% at a sample flow rate of 20 L/min [24]. In 2022, Han et al. developed a new battery-operated stationary electrostatic bioaerosol sampler with a high concentration rate of ∼5 × 10^4^ min^−1^ with 0.2 mL elution liquid, and the sampler achieved ∼50% collection efficiency with 0.2 mL elution liquid [25].

Electrostatic sampling methods offer several advantages; however, concerns exist regarding the use of electrostatic precipitators for bioaerosol sampling. One major disadvantage of ESP is its low collection efficiency at high sampling flow rates. Several studies have shown that ESP has minimal impact on the culturability and viability of microorganisms after four hours of sampling. Despite significant developments in this field, there are only a limited number of commercial electrostatic samplers available for bio sampling, which remains a major concern [11].

#### 2.1.3. Passive Sampling through Thermal Precipitation

Thermal precipitation is one of the oldest and most attractive sampling techniques for the collection of airborne particles owing to its high collection efficiency [26]. The thermal precipitator works mainly based on the principle of thermophoresis. When a thermal field exists between a heated and cooled field, it produces a force on the airborne particles, and moves the particle from hot surfaces to the cooled surface, helping to deposit it over the cooled surface. Under properly regulated and controlled conditions, all the airborne particles from the sample air can be easily deposited on the cooled surface, and this basic phenomenon is known as thermophoretic motion. The deposit can be examined by microscopic studies without any further sample preparation. Thus, thermal precipitation is the most ideal sampling technique for dust sampling, smokes, fumes, and also for radioactivity monitoring [26]. 

Generally, the hot surface of the precipitator can be heated up to 125 °C, while the collecting surface is cooled below room temperature (approximately 25 °C) by circulating water as a heat exchanger. If the sample is collected over filter paper, the deposits can be transferred to regular agar plates and examined after incubation. However, if glass microscopic cover slips are used as the cooled surface for sample collection, immediate microscopic study is needed [26]. 

The thermal precipitator for dust sampling was first described by Green and his workers in 1935. Later, Kethley and his co-workers used thermal precipitators for aerobacteriology, and Wright used a gravimetric thermal precipitator as a dust collector for animal inhalation experiments in his lab [27]. Studies have shown that thermal precipitators have better efficiency when the airborne particle size is below 5 microns, and the collection efficiency decreases if the air velocity is high. Thermal precipitators also help to study the particle size distribution of airborne particles. Mossop and Tuck-Lee used a thermal precipitator to study the composition and size distribution of airborne particles produced by the burning of silver iodide and sodium iodide in acetone [28]. Du Toit and his co-workers used three different samplers, including a konimeter, Dosimeter, and thermal precipitator, for simultaneous airborne dust sampling in eight asbestos mines in 1977, which helped to analyze the different relationships between each type of asbestos [29].

Kasper slightly modified the traditional thermal precipitator in 1981 to obtain a well-defined aerosol flow and to reduce the loss of fine airborne particles. After 1990, many modifications were introduced in the traditional thermal precipitator to obtain better collection efficiency. Tsai and his co-workers developed a modified thermal precipitator with two plates and a thermal insulating shim to separate them, maintaining a high and uniform temperature gradient throughout the experiment [30]. Maynard developed a new type of thermal precipitator which was specially built to analyze and collect the ultrafine aerosol particles on the supporting grids of a scanning transmission electron microscope. The analysis results showed that the particle distribution on the microscope grid was found to be uneven on a millimeter scale, whereas it was relatively even on a micrometer scale [31].

In the 21st century, researchers have developed new and advanced thermal precipitator for aerosol sampling. Wang and his co-workers built a new thermal precipitator with a cylindrical configuration and a size-selective inlet and examined its performance using the differential mobility analyzer (DMA). The collection efficiency was found to be negligible for particles with diameters ≤ 300 nm [32]. In the subsequent year, again, they developed a new type of disk-to-disk thermal precipitator. It also achieved the same results as their previous studies [33]. A group of German researchers developed an advanced type of thermal precipitator for the deposition of airborne particles over living cells. They designed their thermal precipitator with two parallel plates, and it was particularly customized for the exposure of cells to nanoparticles. The results were not good, and more optimization steps were required to obtain better results [34]. 

Overall, studies have shown that thermal precipitators are best suited for studying smaller-sized particles and for determining the size distribution of airborne particles. Although the collection rate is low in the range of 7 cm^3^ min^−1^ to 1 L min^−1^, thermal precipitators have the advantage of low pressure drop and do not require a vacuum source. Thus, future works are required to increase the collection efficiency of bioaerosol samples of large particles. 

### 2.2. Active Sampling

Active sampling methods are used to quantify the concentrations of bioaerosol samples. Pumps or fans are utilized in active sampling methods to collect gases and vapors from the environment at certain flow rates and deposit them over a sampling medium. Furthermore, particle size selection and hygroscopic growth can also be accomplished in active samplers by pre-conditioning their air streams [35]. By using active sample collection, aerosols can be sampled regardless of size, inertia, etc., allowing for the collection of small particles that are missed by passive sample collection. In general, active samplers have more complicated structures compared to passive samplers, requiring constant calibration and sterilization [36]. The culture technique is employed in most of active samplers as a step to analysis. This is a traditional method for sampling and analyzing bioaerosols that is time-consuming, less sensitive, and expensive. This is because, in the culture method, agar plates are used as the collection media, and these plates then have to be further incubated and then analyzed to quantitatively determine the bacteria, virus, fungi, and other matter. Sometimes, it is observed that the sample collected in the agar plates gets damaged by the sampler itself. So, it is important to proceed with a proper plan after agar plate-based collection to achieve complete sample recovery. 

#### 2.2.1. Impaction

In the sampling of bioaerosols, impaction-based samplers are the most used method. Typically, impactors have a target and a series of circular or slotted nozzles. Bioaerosol impactors sometimes use multiple impaction nozzles, and these impactors are known as multi-nozzle impactors. Commercially available impactors differed according to a number of factors, like the number of nozzles, the flow rate(s) used, and the number of collection stages [37]. In this format, the inertial forces of different types of particles are utilized for the collection of airborne particles. For example, air flows are collected by samplers, and the particles with high inertia are deposited over agar plates using centrifugal force [38]. Thus, the collection efficiency of any sampler is predominantly dependent upon the density and diameter of the airborne particles as well as the nature of the collection stage. Agar plates, glass slides, and also other solid surfaces are commonly used for the collection of microorganisms and subsequent microscopic analysis [39].

Impactors have multiple advantages when compared to other bioaerosol sampling methods because of their user friendliness and convenience. Additionally, they are considered as the best choice for the collection of culturable microorganisms, and agar plates are one of most commonly used collection surfaces in impaction-driven samplers because once the samples are collected over the agar plate, they can be directly transferred into the incubator for further analysis without any other additional intermediate steps. Additionally, the microorganisms collected over agar plates can be easily scarped with water, and one can examine them using standard molecular methods such as polymerase chain reaction (PCR). An experimental cutoff size of d_50_, which represents the particle diameter with 50% collection efficiency, is the most important parameter for the impactor, and a lower cutoff size allows for the capture of smaller bioaerosols [39]. Agar plates have several advantages as a method for bioaerosol collection. However, they also have some limitations, including the potential mixing of multiple colonies, longer sampling runs that can lead to agar plates drying and affecting cell viability (which can be mitigated by spreading mineral oil onto agar plates), sample loss when different collection surfaces are used, and impaction that can damage membrane integrity and the viability of airborne microorganisms. Despite these limitations, agar-based impactors remain popular for bioaerosol collection due to their ease of use and availability of reference information [40].

There are many different types of commercially available impactors. The single-stage and seven-stage ultimate Andersen impactors, BioStage impactor, AeroTrap^TM^ Total Fungal Sampler, and BioImpactor 100-08 (AES) are just a few examples [40,41]. From the years 1920 to 1945, many development studies were carried out to design the impactor. In 1945, the Cascade impactor, which provides information about particle size distribution, particle-laden air flow, impaction plates, and the concentration of aerosols, was first developed. In 1956, a new Andersen impactor-type cascade impactor was developed specifically to study the viability of bioaerosol particles. Andersen impactors consists of six stages and have 400 nozzles. Later, different types of handheld Andresen impactors were developed and used with external air flow devices for working in places where electricity is limited or not accessible [42]. In early 2000, Lee and his co-worker developed a new type of impactor with a cooled impaction plate for the simultaneous detection of *Escherichia coli* and *Bacillus subtilis*. The usage of a cooled pad without any coating material significantly reduces the particle bouncing performance. The collection efficiency was increased to 10–15% when the impaction plate was cooled below 10 °C [43]. 

Recent studies have mainly focused on the development of portable, battery-operated impactors for bioaerosol collection without the use of external heavy pumps or fans [44,45,46]. Zhen and his co-workers evaluated the biological collection efficiency of the portable BioStage impactor and the Reuter centrifugal sampler (RCS) by measuring the airborne bacterial and fungal concentrations in various places in China at flow rates of 28.3 and 100 L/min, respectively, for a sampling time of 50 min in each environment. The collection efficiency of each impactor was analyzed by qPCR, and the BioStage sampler had shown better cell culture, whereas the RCS sampler had less particle bounce and a shorter sampling time [47]. Park and his co-workers designed a new single-stage virtual bioaerosol collector by using the micro-electro-mechanical systems (MEMS) process. The physical performance of the impactor was evaluated using atomizer-generated polystyrene latex (PSL) particles, and their size distribution was analyzed using a aerodynamic particle sizer and a scanning mobility particle sizer. The cut-off diameter size was measured as 0.95 μm, and it was well matched with the flow simulation. The collection efficiency was further verified with *Staphylococcus epidermidis*, and the study’s results proved that the developed virtual impactors were most suitable for viable microorganisms [48]. Chen et al. tried to develop a new high-flow bioaerosol impactor (HighBioTrap) for the rapid detection of microbes. This portable device has a high sampling flow rate of 1200 L/min with an impaction velocity of 10.2 m/s, and its performance was tested in both laboratory and natural environmental conditions. The BioStage impactor was used as a reference sampler to assess the performance of the HighBioTrap. The designed system showed low physical collection efficiency due to a higher desiccation effect, but high flow rates enabled the detection of low-level pathogens [49].

Currently, impaction-based samplers are commonly used to collect bioaerosols due to their low cost, easy handling procedure, and the fact that they do not require any additional post-processing steps. On the other hand, impaction-based samplers have a few limitations: (1) the collection efficiency can be reduced due to particle bounce effects, (2) applying shear forces to bioaerosols decreases their bioactivity, and (3) the overlapping of microorganisms affects bacterial colony formation [40,41].

#### 2.2.2. Cyclone

The cyclone sampler works on the principle of centrifugal force. Rotating the air flow inside the chamber creates the centrifugal force and separates particles of interest from the flow. Then, the separated particles can be collected at the bottom, while the particles with a size that is less than the specified size remain in the air stream and are deposited over the pre-weighed filter medium [50,51]. The introduction of a liquid medium to the chamber wall is frequently carried out to enhance the collection efficiency and viability of bioaerosols [52]. While the flow speed and the size of the bioaerosols are the main parameters of the centrifugal forces, the optimization of the cyclone’s geometry and operating conditions, including the size and orientation of the inlet, aerosol size and density distribution, particle bounce properties, and the conductive properties of the cyclone, is generally required to maximize performance [52]. Thus, over the past few decades, a vast number of studies have been carried out for the optimization of cyclone samplers with high collection efficiency. 

The size of the vortex finder is an important geometric component of cyclones, and numerous research works have investigated the effect of vortex finder properties on collection efficiency [51,53]. Brar et al. conducted a study on the effect of the vortex finder diameter on the flow rate and collection efficiency of cyclone collectors using computational fluid dynamics. They investigated five different vortex finder diameters and found that a decrease in the vortex finder diameter led to an increases in pressure drop and collection efficiency of 47.84% and 9.54%, respectively, while an increase in the vortex diameter reduced the pressure drop by 23.87% and the collection efficiency by 7.70% [54]. Elsayed and his colleagues conducted similar research, studying the effect of vortex finder dimensions (length and diameter) on collection efficiency and flow field pattern in nine different cyclones using large eddy simulation (LES). They varied the vortex diameter from 0.3 to 0.5 times the cyclone diameter and found that a 40% decrease in the vortex finder diameter increased the collection efficiency to 175%. The maximum tangential velocity was achieved when the vortex finder diameter was decreased [55]. Other studies have aimed to increase collection efficiency and reduce pressure drop by varying the exit pipe dimensions. In one study, a comparison of experimental and numerical results showed that an increase in exit pipe dimensions helped to decrease pressure drop but did not affect the collection efficiency of the cyclone [56]. 

In the 1990s and early 2000s, many research works attempted to increase collection efficiency by altering their physical size and properties, but in recent years, many researchers have tried to develop new types of cyclone samplers using advanced features. Wet cyclones have received a lot of interest in contrast to conventional cyclones because of their improved sample collection efficiency. King et al. developed a batch-type wetted wall bioaerosol sampling cyclone (BWWC) and studied its biological efficiency and sample retention properties. The sampler was designed to work at an air flow rate of 400 L/min and concentrated the particles into 12 mL of water. The collection efficiency and sample retention were evaluated using polystyrene latex (PSL) beads, sodium fluorescein/oleic acid droplets, and *Bacillus atrophaeus* spores. The sample retention was calculated to be 90% and reduced to 10% after 8 h, and the collection efficiency was calculated to be 50–60%, 1.5%, and 35% for the PSL beats, oleic acid droplets, and spores, respectively [57]. The same type of wet cyclone was developed by McFarland and his co-workers. The sampling flow rate was fixed at 1250 L/min, and the liquid flow rate was fixed at 1 mL/min. The results showed that the developed cyclone sampler has a low collection efficiency, but liquid consumption and pressure drop was improved [58].

Wet cyclones are also used for the real-time detection and continuous motoring of bioaerosols. Cho and his co-workers developed a new type of wet cyclone sampler for the collection of microorganisms. The newly developed Automated and Real-time Bioaerosol Sampler based on Wet-cyclone (ARBSW) was used to collect microorganisms in a liquid medium. The results proved that the ARBSW had superior collection efficiency and excellent particle transfer efficiency. This system also showed better results for the quantitative characterization airborne particles when integrated with a microfluidic flow cytometer. This system achieved > 95% collection efficiency for *Staphylococcus epidermidis* and *Micrococcus luteus* [59]. Similarly, Sung and his co-worker developed a new type of in-line wet cyclone sampler for the early detection of airborne viruses. This model consisted of four different sections: a pre-separator stage, impactor-stage collection, a fluidics system, and a virus sensing stage. Thus, all virus detection steps, such as air sampling, hydration, drying, and assay works, were carried out in a single system without any pre- or post-treatment. This system showed 100% collection efficiency for large-sized PSL beads, and it showed better collection efficiency for airborne viruses such as H1N1 and H3N2 [60]. 

Heo and his co-workers designed a novel, simple, and highly efficient wet cyclone sampler for the COVID-19-causing virus. They integrated continuous aerosol-to-hydrosol transfer (ERC-ATHT) into the wet cyclone sampler so that it could effectively collect bacteria of less than 300 nm and enhance the concentration to 2.4 × 10^6^ in a few minutes. The collection efficiency was satisfactory under both laboratory and real environmental conditions [61]. Similarly, Lee and his co-workers developed a new type of cyclone–cytometer-integrated air monitor by integrating a wet cyclone air sampler into a DC impedance microfluidic cytometer. The wet cyclone sampler collected the airborne samples and concentrated it in 10 mL of aqueous solvent, and the samples were detected by the microfluidic cytometer. The sampling efficiency of the cyclone sampler was calculated to be 28.04%, and the detection efficiency was calculated to be 87.68%, which produced a total efficiency of 24.59%. Moreover, this design can also be used for the detection of specific species with proper antibody fluorescent labeling [62].

In another work, Li et al. designed a new robot-assisted highly portable cyclone sampler for the detection of both bacteria and viruses, especially COVID-19. The sampler was named Yao-CSpler, and its collection performance was studied using uniform aerosolized polystyrene (PS) microspheres, *Bacillus subtilis var. niger*, and *Pseudomonas fluorescens* in both indoor and outdoor conditions. The cutoff diameter of the sampler was experimentally calculated to be 0.58 µm. Comparing the performance of the developed sampler with the traditional BioSampler (SKC Inc., Dorset, UK), the developed sampler showed better performance in terms of bacterial diversity, and COVID-19 detecting performance was assessed by performing experiments in both Wuhan and Beijing during COVID-19 outbreaks. The Yao-CSpler was able to collect COVID-19 with a detectable concentration level of 9–219 viruses m^−3^ [63].

Several studies have also shown the effectiveness of using cyclone samplers for bacterial sample collection. King and his co-workers studied the cell culturability and DNA integrity of *Escherichia coli* using a 300 L/min wet cyclone sampler and 800 L/min inertial impactor at two different temperatures. Compared with the inertial impactor, the wet cyclone sampler had a 100-fold higher collection factor at room temperature and 4000-fold higher collection factor at 42 °C. Pulsed field gel electrophoresis (PFGE) and photographic evidence were used to study DNA integrity, and the study results showed that molecules larger than 500,000 base pairs were identified in the wet cyclone-collected microorganisms [64].

Duquenne et al. carried out another study with *Escherichia coli* to evaluate the collection efficiency of the BC-112 NIOSH cyclone for the measurement of endotoxins under laboratory conditions. The BC-112 NIOSH cyclone is type of a personal sampler which contains a main metallic body with a 2 mm inlet, one collection stage with a 1.5 mL collection tube, and 37 mm of three pieces of cassette as final collection stage. They validated the endotoxin collection performance of BC-112 NIOSH by modifying the PVC filter, used as a collection medium, and endotoxins were directly extracted over the cassette. The collection efficiency of the modified sampler showed better performance than a close-faced cassette collector [65]. 

Numerous research studies are currently underway for the simultaneous collection and detection of airborne particles. Cyclone samplers are popular due to their simple construction, high reliability, high collection efficiency, and ability to operate at wide temperature ranges. They also have low maintenance costs compared to other sampling methods, as they do not contain any moving parts. Additionally, cyclone-based samplers are ideal for collecting viable microorganisms. However, there are limitations to cyclone samplers. The shear forces in the liquid medium can reduce the bio-efficiency, which is the ability of the sampler to maintain the viability of bioaerosols during and after the sampling process. Evaporation of the collection medium can also result in some collection losses. Moreover, the size and flow rate of each cyclone may vary due to differences in cyclone geometry and air flow, which can impact the collection efficiency. Cyclones can be used for large volumes of air samples, and miniature cyclones can be used as personal samplers in highly hazardous environments. In some cases, cyclone samplers are used as pre-classifiers for the removal of larger particles in the airstream [43]. Similar to impactors, cyclones are commercially available in the market. 

#### 2.2.3. Impingement

The impingement technique for bioaerosol sampling is almost similar to impaction- and cyclone-based approaches. In the above techniques, samples are collected over dry solid surfaces or by a filter; this provides a chance to reduce the viability of microorganisms during desiccation. Thus, impinging-type samplers use liquid media for sample collection. When the air stream flows down through the nozzles, it enters into the liquid medium with high velocity. Moreover, impingers also utilize particle inertia for the sample collection. Similar to impaction-based methods, the inlet characteristics of the sampler strongly influence the collection and recovery efficiency at high flow rates. Thus, similar to impactors and cyclone samplers, the curved inlet is used to prevent larger air particles entering into the sampler [53]. 

The major drawback of using a liquid medium is the fact that a high air velocity agitates the liquid and induces foam formation in the liquid. This strongly affects the viability of culturable bacteria. This problem can be avoided by the addition of antifoam agents and some kind of protein molecules. Additionally, antifreeze agents are also added for the resuscitation of bacterial cells. This could also prevent collection fluid loss and decrease cell damage. Moreover, the microorganism can be subjected to osmatic stresses during sample collection, and this can be solved by varying the liquid medium for each sampler [40].

Available commercial impinger samplers include the All Glass Impinger 30 (AGI-30), the Burkard multistage sampler, the SKC Biosampler, the multi-orifice impinger (MOI), the modified personal impinger (MPI), and the multi-stage liquid impinger (MLI) [37]. In general, impinger instruments are made of glass; therefore, they have the advantage of being less expensive, but their robustness is a concern. The most commonly used liquid impinger is the “Biosampler”. This model consists of an air inlet, three tangentially arranged nozzles, and a collection vessel. In addition, the Biosampler also contains a pump which is capable of extracting air particles from the air stream at a flow rate of 12.5 L/min with a sampling time of 0.5 to 4 h. During the sampling process, the air stream which contains the bioaerosols is directed towards the wall of the sampler, where a liquid film is formed by the centrifugal motion of the liquid. During operation, the liquid spins upward and removes the collected particles from the sampler’s inner wall [54]. 

Conventional impingers use only water or liquids with almost the same viscosity as water, whereas the BioSampler (SKC) uses buffer or highly viscous collection fluids as collection media. Zheng and his co-workers studied the collection efficiency of the SKC BioSampler for size-resolved viable airborne particles using an ultraviolet aerodynamic particle sizer. The experimental study results showed that the collection efficiency decreased from 82.7% to 24.8% at V_cl_ of 20 mL and 5 mL, respectively, at a constant flow rate of 12.5 L/min. The results were further confirmed by the BackLight DNA stain method. One of the main drawbacks of the SKC BioSampler is its low rate of capture of viable bioaerosols, which is a result of high sampling velocity as well as the intense motion of the collection fluid. This decreases the viability of culturable bacteria. Many research works have proved this [66].

In addition to the BioSampler, the All Glass Impinger with 30 mm nozzles (AGI-30) and the Greenburg–Smith impinger are the most used impingers for bioaerosol sampling. They operate at a flow rate of 12.5 L/min and 28.3 L/min, respectively. Both impingers are used to collect the total microorganisms in the environment, but some researchers have reported that they have low sampling efficiency and experienced high particle loss during the collection process [53,54].

Typically, the characterization of airborne bacteria in an outdoor environment can be quite challenging because of their dilute nature. To overcome this, many investigators have been using impingers for outdoor sample collection, and it is necessary to develop systems with a rapid and reliable approach for viable cells, especially in outdoor environments. Jang et al. developed a high-volume impingement sampler for the collection bacterial cells from a wastewater treatment plant (WWTP). A Cytosense flow cytometer (Cytobouy) was used to evaluate bacterial cell density, and its results were compared to quantitative PCR (qPCR). The regression analysis of this work showed that the evaluation of bacterial cell densities in both methods are almost the same, and the results proved that the proposed approach was most reliable for the estimation of bacterial cell densities in an outdoor environment [67].

In studies on airborne bacteria, the sampling strategy is crucial and should not affect their viability, culturability, integrity, and metabolic activity. Thus, it is very necessary to develop an effective biosampler with high sample collection efficiency. Santl-Temkiv et al. characterized a high-volume impinger (Karcher, Alfred Kärcher GmbH & Co. KG, Winnenden, Germany) to study the physical properties of airborne bacterial cells. The developed system showed better collection efficiency, and the collection efficiency increased with an increase in particle size, and the cut-off diameter was calculated to be between 0.5 µM and 1 µM. In addition to collection efficiency, the system showed better retention efficiency even after a sampling time of 120–300 min. Upon comparing the results with another four commercially available impingers, the particle loss was found to be significantly diminished [68]. 

In recent years, microfabrication technology has received greater attention for the development of minimized systems for bioaerosol sampling. Mirzaee et al. designed and fabricated a microfluidic impinger (microimpinger) for airborne particle collection. This system controls bubble generation and foam formation by passing the air through microchannel arrays. The microchannel arrays were fabricated by using a technique called soft-lithography, and PDMS was used to control the air leakage in the microchannels. A collection efficiency of 90% was recorded when studied the different-sized fluorescent polystyrene latex particles on the polycarbonate filters. A numerical stimulation developed by CFD was used to understand the mechanism of bubble formation during sampling, and this system can be also used to study the bubble size effect during the sampling process [69].

Vives et al. used an impinger for the quantification of SARS-CoV-2 in bioaerosols. They developed a new system by integrating a liquid impinger with ddPCR. The developed system showed a bio-efficiency of 44.6%, and the collection efficiency increased by 50% following an increase in sample air volume from 339 L to 650 L, but when comparing the detection limit of this system with RT-PCR, it showed an efficiency value that was five times smaller [70]. Habibi et al. also demonstrated a simplified protocol to collect SARS-CoV-2 in bioaerosols by impinging through a series of three glass bottles [71].

The development of highly efficient impingers is still in its nascent stage. Liquid impingers have some major drawbacks. Firstly, many liquids used in impingers can easily evaporate, resulting in viability and particle loss. Secondly, the use of high-density liquid can sometimes affect the collection efficiency of light-density microorganisms. Thirdly, some research studies have reported that high-velocity air can damage or destroy some bacteria, further impacting the collection efficiency of impingers. Therefore, for the collection of airborne fungal spores and bacteria, impactors or cyclones are suggested for most environments rather than impingers. Impactors and cyclones are less affected by these limitations and are able to achieve high collection efficiency for these types of particles [40,41].

#### 2.2.4. Filtration

Filtration is a commonly used method for capturing bioaerosol particles due to its ease of handling and low cost. Porous membrane filters are used in the filtration method to collect microorganisms by passing air through them. There are two ways to analyze the airborne particles collected using filtration. Firstly, the particles deposited on the filter can be directly examined using a microscope or electron microscope. Alternatively, the deposits of bioaerosol particles collected on the filter can be eluted as a liquid and analyzed using different techniques. The filters used for this technique are typically made of materials such as glass fiber, polyvinyl chloride (PVC), polycarbonate, mixed cellulose acetate, polytetrafluoroethylene, and nylon, but other types of filters can be selected based on the analysis techniques [38,39]. 

In the early 1980s, commercially available sampling devices utilized various techniques to capture airborne microorganisms, and the collection process involved using agar gels (such as impactors/slit samplers) or liquids (such as impingers/cyclone-based samplers). These samplers were primarily designed for use in hospitals, offices, and other indoor environments. However, if the concentration of microorganisms in the air exceeds 10^4^ microorganisms/m^3^, these samplers cannot be used in the environment without further modification. This is because high concentrations of microorganisms would make it impossible to count colony-forming units (CFUs). To address this problem, numerous investigations were conducted to develop new types of filters [40,42].

Zimmermann and his co-workers were among the first to investigate the trapping efficiency of membrane filters for bacterial populations [72]. In 1984, G. Blornquist and co-workers analyzed improved techniques for sampling fungal airborne particles in highly contaminated environments. They developed a new Nuclepore filter made of polycarbonate for collecting airborne particles and compared its collection efficiency with a modified slit sampler and personal cascade impactor. The results of their study showed that the new Nuclepore filter and modified slit sampler had similar trapping efficiencies in the range of 10^3^–10^8^ colony-forming units. The slit sampler was limited to stationary emission studies, whereas the Nuclepore filter method was the most suitable. Furthermore, the personal cascade impactor and Nuclepore filter showed the same trapping efficiency in all microorganism ranges. However, the cascade impactor required more time for equipment preparation for airborne sampling, which was a major disadvantage. Therefore, the study concluded that Nuclepore filters were more efficient and also provided information on size distribution [73]. After this study, Pasanen et al. used both filtration and impactor methods simultaneously to study airborne fungal spore concentrations, and total and viable spore counts were analyzed using a scanning electron microscope (SEM). Their SEM study results showed that the filter method is more suitable for measuring spore levels above 10^3^ spores/m^3^, whereas the impactor method is more suitable at spore levels below 10^5^ spores/m^3^ [74].

In the 1990s, several new types of filters were introduced for bioaerosol sampling. In 1990, a group of Swedish researchers utilized a polystyrene filter monitor loaded with polycarbonate or cellulose acetate filters for heavily microorganism-contaminated working environments. Microscopic tests such as scanning electron microscopy and Light Microscopy (LM) were used to estimate the concentration and morphology of microorganisms [75]. In 1994, Throne and colleagues compared three different sampling methods for studying viable microorganisms in 25 diary barns during two seasons: summer and winter. Among the three different filters, the Nuclepore filtration method, using air filtration with subsequent elution and culturing, showed better performance for molds and thermophilic organisms, whereas the glass impingement method exhibited excellent results for yeasts and mesophilic bacteria concentration [76].

W-H Lin and C-S Li evaluated the performance of impingement and filtration methods for yeast bioaerosol sampling. They used gelatin-type filters in addition to the AGI-30 impinger and a Nuclepore filter. The study’s results showed that Nuclepore and gelatin filters have <20% total recovery. Among the different types of filters, the gelatin filters exhibited better sampling performance than the other filters [77]. Later in the 1990s, Nasman and his co-workers used a new type of filter consisting of mixed cellulose acetate and nitrate soaked in glycerol for passive sampling. They studied the sampling performance of this new type of filter for three different fungal species and also compared its performance with closed-face polycarbonate filters. The glycerol-soaked filter exhibited a better correlation with polycarbonate filters with regards to the total spore count [78].

Most of the results of filtration studies show that the filtration-based samplers can generally be used as personal samplers. This is because, when the filtration method is used in highly contaminated environments, the enumeration of bioaerosol becomes more difficult, and at the same time, the drying of microorganisms from the filter after collection is also a difficult task because some of the fungi and bacteria are still alive in the filter’s pores. Moreover, sampling time and relative humidity plays a major role in filter-based sampling. For example, when the temperature exceeds 30 °C and relative humidity increases from 30 to 85%, many bacterial cells become non-viable. In the year 2001, Wang and his group members studied the effect of sampling and relative humidity on the bio-efficiency of two different personal samplers against five aerosolized microorganisms (fungal spores, endospores, and bacterial vegetative cells). The culturability of the endospores decreased with an increase in sampling time and relative humidity, and the microorganisms extracted required vortex or ultrasonic agitation. In addition to relative humidity and sampling time, the effect of storage is also an important factor for the recovery of captured airborne particles. An increase in the storage time also decreases the bacterial colony recovery rate [79]. 

Groups of researchers from Kanas State university evaluated the concentration of bioaerosols in a swine barn by both filtration and impaction sampling. From their results, membrane filtration sampling can be used only for qualitative surveys, while the impaction sampler was more suitable for both qualitative and quantitative sampling [80]. After the severe attacks of anthrax in 2001 and SARS in 2003, and the long-lasting attack of avian flu and influenza virus H1N1, more public awareness was created for the people to protect them from these kinds of natural bioaerosols. Thus, many researchers integrated nanoscale technologies with filtration methods, and they introduced nanofibrous filtration as a promising alternative for existing filters because nanofibrous filter membranes have small pore sizes and large surface areas. Moreover, traditional filtration membranes only help to capture microorganisms, and they do not provide sufficient protection against the above-mentioned kinds of pathogens. Thus, it becomes necessary to upgrade the traditional filter to collect pathogens without harming the person operating it. As the samples are expected to be viable for a better analysis, the filter process should be designed in a way that ensures the collection of the maximum number of samples without causing damage, as well as the quick removal and storage of samples with minimum harm to the handling person. Further, the exposure time after sample collection from the filter is reduced significantly because the detection of bioaerosols requires all collected samples (without any losses) to have a precise detection limit. For this, people are now developing existing systems in hybrid forms including latest technologies. 

Zhang et al. developed a new microwave-assisted nanofibrous air filter using Polyacrylonitrile (PAN) nanofibers using an electrospinning process, and this microwave assisted nanofibrous filter was demonstrated to be an effective approach for pathogen disinfection [81]. Details of already available electrospun nanofibrous membranes for the filtration of bioaerosols and their improvement strategies for the efficient monitoring and detection were discussed in a recent report [82]. Exploiting the antimicrobial effect of silver nanoparticles over activated carbon fibers on contaminated surfaces is considered as an effective control method for improving indoor air quality, but more research works are required to optimize the concentration of silver nanoparticles over electrospun carbon fibers [83]. 

In the 21st century, many nanoparticles designed to be deposited over carbon and cotton filters have been developed to control microorganisms, and they have shown better performance in the deactivation of pathogens. In most research studies, zero-valent silver, iron, TiO_2_ and its nanocomposites, silver nano and its nanocomposites, zinc, carbon nanotubes, and other different types of organic polymer-based nanofibers and nanofillers have been used. These kinds of nanofibrous and nanoparticle-doped filters definitely play an important role in preventing the further spread of disease-causing pathogens [84,85]. 

#### 2.2.5. Other Approaches

Recently, microfluidic technology has been incorporated into bioaerosol samplers. Microfluidic chips are known for their miniature size, low cost, high surface-to-volume ratio, and easy integration with other bioaerosol sampling methods. They have been extensively used in various fields, such as food safety, environmental monitoring, and clinical diagnosis [86]. There are several types of microfluidic chips that have been continuously modified for bioaerosol sampling. These include herringbone-based, centrifugation-based, and droplet-based microfluidic chips. In some recent studies, microfluidic chips have been combined with traditional sampling methods to improve their sampling efficiency and develop inexpensive and portable methods [87,88]. In 2016, Bian and his co-workers designed a new microfluidic air sampler for the efficient collection and identification of a mixture of three different bioaerosols: *Vibrio parahaemolyticus*, *Listeria monocytogenes*, and *Escherichia coli*. They used a three-loop double-spiral microchannel microfluidic chip with a combination of herringbone and sawtooth wave-shaped structures to improve the surface area for the accumulation of bioaerosol. This design achieved a sample efficiency of 99.9% within 30 min [4]. Another type of microfluidic chip was developed by Jiang and his co-workers for the rapid capture and analysis of airborne *Staphylococcus aureus* in a hospital atmosphere. The complete analysis process only took 4 h and 40 min, with the limit of detection being 27 cells. Microfluidic chips have the ability to collect bioaerosols such as viruses ranging from 20 nm to 300 nm, as well as 500 nm to 2 µm bacteria. Furthermore, this process did not require any DNA purification process and can be directly used in hospital settings for clinical airborne pathogen sampling [89]. 

The use of droplet-based microfluidic chips has opened up new possibilities for bioaerosol sampling. These chips have many advantages, including their compatibility with most biological and chemical reagents, their ability to serve as microreactors for particles ranging from a few nanoliters to femtoliters (10^−15^ to 10^−18^ L), their short analysis time, their compact and portable design, their low cost, and their high resolution and sensitivity. However, there are also drawbacks to using droplet-based microfluidics. One major challenge is the difficulty of producing monodisperse droplets, and another is the inefficiency in collecting bioaerosols of different sizes. 

Continuous flow-based microfluidics allows for a better control of flow characteristics, but droplet manipulation and rapid detection present complex challenges in continuous-flow droplet microfluidics. To address this, Choi and his co-workers developed a novel microsampler for continuous aerosol sampling using inertial microfluidics that converts aerosols into liquids [90]. They assessed the physical particle efficiency of the microsampler using standard polystyrene latex (PSL) particles ranging in size from 0.6 to 2.1 μm and achieved a collection efficiency of 98% with a microfluidic air flow of 0.6 L/min.

The integration of microfluidic chips with conventional bioaerosol sampling methods has emerged as a modest, portable, and inexpensive method for bioaerosol sampling. Lee and his co-workers reported an interesting study where they developed a new type of air sampler by integrating a wet cyclone air sampler with a DC impedance microfluidic cytometer. The sampler was tested with microbeads, dust, and *Escherichia coli*, and the sampling efficiency was found to be 28.04% for the wet cyclone air sampler and 87.68% for the DC impedance sampler, with an overall efficiency of 24.59%. While microfluidic-based sampling methods have many merits compared to traditional sampling methods, their low flow rate and small sampling volume remain major disadvantages of microfluidic-based samplers [62]. A very recent review on microfluidics-based sampling and detection was provided by Lee et al., explaining all the effective parameters to consider for the on-site sampling and detection of bioaerosols [91]. Interestingly, a typical aerosol-to-hydrosol (ATH) sampler, combined with a microfluidic chip, coated with concanavalin A (ConA) has been developed for the PCR-based rapid detection of airborne coronaviruses and influenza viruses [92]. The enrichment capacity of the ATH sampler was 30,000-fold for both viruses, whereas there was an 8- and 16-fold enrichment for the ConA-coated microfluidic chip for the coronaviruses and influenza viruses, respectively.

## 3. Sampling Efficiency Improvement Strategies

Sampling efficiency is an important factor in the bioaerosol sampling procedure in terms of data interpretation and quality evaluation. Bioaerosol sampling errors can be divided into multiple categories: the heterogeneous distribution of bioaerosols, samples with a restricted number of discrete particles, and innate instrument errors. However, there are several controlling factors that may affect the efficiency of bioaerosol sampling [41,42]. Sampler selection is one of the most important factors for bioaerosol sampling. Very recently, a group of scientists studied six different samplers’ performance for bioaerosol detection in a specially customized wind tunnel with wind speeds of 2–20 km/h [93]. After 10/60 min of sampling, the results implied that the AGI-30 and BioSampler impingers are good for short-time sampling only, meaning that they do not allow for the collection of low-concentration samples. However, SASS 2300 and BSA-350 wet-wall cyclones have higher microbial sampling cultivability in short-time and long-time sampling, but they fail to provide quantitative measurements of aerosols. Also, to maintain the reduced microbial activity of the collected aerosols through polycarbonate filters, gelatine-type filters were recommended by the authors of the study. Thus, the sampler type will also influence the detection results. Before selecting an appropriate sampler, one should consider the type of bioaerosol to be analyzed, the type of analysis they will use, bioaerosol sample size, sample concentration, and sample biomass. Conversely, if the biosampler has already been selected, then other sampling parameters are prepared to fit the sampler type. However, the second approach is not advisable in most cases of bioaerosol sampling. The ease of cleaning in between runs is essential for minimizing sample contamination [94].

Then bioaerosol collection medium is one of the key factors in bioaerosol sampling. One should use the amount and type of collection medium recommended by the sampler’s manufacturer or sampler’s developer; otherwise, the sampling efficiency and sampling time will be affected. Collecting media commonly used in bioaerosol sampling include deionized/autoclaved water, sodium chloride solution, and phosphate-buffered saline (PBS) with or without surfactants like Tween-80, Tween-20, and Triton X-100, all of which are used in order to provide a neutral environment during the sampling process, and mineral oil is considered as the best option for qPCR [38].

Sampling duration and sampling frequency are the most essential elements in any bioaerosol sampling process. The sampling duration is mainly dependent on the bioaerosol particle size, concentration, collecting medium, and type of biosampler used in the study. The use of filters can lead to long sampling durations, which cause damage to the bioaerosol particles, and a long sampling period can lead to the overestimation of concentrations because too many spots will be occupied before sampling is finished in multi-nozzle impactors [41,42]. Research is still needed to determine whether sampling duration affects passive sample quality and representativeness.

The quality control and assurance of any bioaerosol sampling process is best assured using blanks or controls. When agar plates are used for sample analysis, some of the blank plates are kept aside for incubation. If any small observable change occurs in the blank means, it can be subtracted from the actual analysis results; otherwise, if the change is comparable with the actual sample results means, the whole batch analysis should be repeated again [38]. The limitations of using culture methods after sample collection, such as the fact that this method may not give pure culture, which will lead to low sample concentrations, are also an important factor to consider. In fact, transportation time, storage temperature, and poor knowledge among the handlers will also affect the culture. Best practices and best-established protocols also help to confirm quality control and assurance. 

## 4. Detection Methods

The detection of bioaerosols is the most important step in bioaerosol monitoring and the effective and efficient quantitative determination of airborne bioaerosols. The basic traditional method of detection is the culture method where after the collection of the samples on agar plates, they are cultured through some type of incubation protocol. This favors bioaerosol growth for analysis. As mentioned above, this method is not suggested because of its many limitations, such as its lack of purity. Apart from that, bioaerosol detection methods can be divided into two major categories: molecular detection methods and immunological detection methods. Molecular detection methods include polymerase chain reaction (PCR), mass spectrometry and sequencing, LAMP, and so on. Radioimmunoassay (RIA), fluorescent immunoassay (FIA), enzyme immunoassay (EIA), and enzyme-based halogen immunoassay (HIA) are considered traditional immunological detection methods, whereas fluorescence and chemiluminescence are some of the emerging immunological detection methods for the on-site detection of bioaerosols [95].

### 4.1. Molecular Detection Method

Molecular detection methods have received considerable attraction in the past few decades. Recently, molecular detection techniques have experienced many developments, but polymerase chain reaction (PCR) is still widely used as a standard method for the detection of bioaerosols in both research and diagnostic laboratories. 

At first, conventional PCR was introduced by Karry Mullis in the year of 1983. Conventional PCR is a system which replicates the specific type of DNA sequence from either a small amount of DNA or RNA in the presence of DNA polymerase and primers under a short period of time. During the nucleic acid amplification process, they follow three different steps. As the nucleic acids of target microorganisms are released from the bio aerosols, they are denatured, annealed, and extended to generate amplicons, whereas the number of amplicons mainly depends on the number of PCR cycles. DNA sample preparation is the most important step in PCR techniques. Each experiment requires different DNA sample preparation procedures. The PCR products are usually analyzed by using gel electrophoresis to confirm whether or not a single product with an exact size is formed [96]. 

Although conventional PCR is a highly sensitive technique for bioaerosol detection and identification, it has some disadvantages. First, there may be non-specific product amplification. Second, the commonly contained compounds in air samples can inhibit the amplification process, so the assessment of environmental interference before PCR is crucial. In this case, the purity of culture of the samples comes into the picture. Finally, conventional PCR cannot differentiate between viable and non-viable pathogenic microorganisms. Also, many PCR devices have a limit of detection of 10^3^ viral genome copies/mL, whereas in the case of an influenza A outbreak, the viral concentration is in the range of 10^3^–10^4^ genome copies/m^3^ of air [92]. To overcome the limitations related to PCR, many metal/metal oxide nanoparticles have been used as PCR enhancers to make the PCR more sensitive or productive. Carbon dots, metals (such as Au and Ag), metal oxide (such as iron oxide) nanoparticles, and inorganic metal alloy nanoparticles are commonly used to increase PCR efficiency by enhancing the binding efficiency of template DNA with DNA polymerase and DNA primers and facilitating thermal cycling [97]. In fact, one research group attempted to develop an aerosol-to-hydrosol sampler with a concanavalin A-coated microfluidic chip to avoid the issues associated with the PCR’s detection limit for viruses [93].

qPCR (quantitative or real-time PCR) is a type of PCR method used for pathogen detection. It is the process of amplifying the bacteria with some specific fluorescent dye reagents, which are called DNA probes. These fluorescent dyes form a covalent bond with the DNA of dead cells so that the PCR can only detect and amplify DNA from living cells. And these DNA probes are more specific to their targets, meaning that multiple targets can be analyzed simultaneously [98]. Chang and his co-worker used a culture assay and qPCR to assess the exposure risk of airborne pathogenic *Legionella pneumophila* and *Escherichia coli*, used as model microorganisms [99]. The results showed that the *Legionella pneumophila* collection efficiency was greater than the *Escherichia coli* collection efficiency by a factor of 2.7–12.2 (*p* = 0.005). Ethidium monoazide (EMA) and propidium monoazide (PMA) were used as DNA probes for the detection of *Legionella pneumophila.* The quantification of viable cells (for low quantities) was reported to be better with droplet digital PCR (ddPCR) than qPCR [100]. Similarly, for low levels of nucleic acids (Cq ≥ 29) and protein contaminants, this technology is considered to be highly precise, providing reproducible results [101].

It is noted that bioaerosols present in indoor air are also a concern for human health, and so knowing their quantities it is of the utmost importance. One specific report focused on the issue of the extensive fungi present in indoor air and their detection via many techniques, including PCR [102]. This report describes the efficacy of the different methods developed so far for fungi detection.

RT-PCR (reverse transcription PCR) is the same as PCR but with reverse transcription as an additional step. Reverse transcriptase (RT), which is a DNA polymerase enzyme, first synthesizes complementary DNA (cDNA) that is used as a standard template for the further DNA amplification process. Primers or random hexamer primers are used in the reverse transcription process. qPCR and RT-PCR are the most advanced types of PCR. These methods have been considered as keystones for the molecular diagnosis of SARS-CoV-2 and are highly specific [103]. It is noted that there is also an inherent limitation to PCR, namely inefficient DNA extraction from microorganisms present in bioaerosols, though this can be overcome by using molecular strategies [104]. In 2021, Mutong et al. reported airborne archaea detection using qPCR and high-throughput sequencing. The concentrations ranged from 10^1^ to 10^3^ copies·m^−3^ (455 ± 211 copies·m^−3^) [105]. The samples were collected using the BioSampler (SKC Inc., Eighty Four, PA, USA), which was equipped with a 20 mL glass vessel filled with PBS. The sampling time was 3 h, and a flow rate of 12.5 L·min^−1^ was used. Another interesting development was put forward by Natasha and coworkers, who collected bioaerosols from cage-housed and floor-housed poultry systems. Stationary area samplers and personal sampling devices were employed. Archaea were identified and quantified with PCR followed by gradient gel electrophoresis and band sequencing [106]. In fact, airborne archaea were collected from swine confinement buildings using 25 mm gelatin filters to capture the inhalable microbial biomass [107]. DNA was extracted and used for PCR amplification of the archaeal 16S rRNA gene. The results suggested that the concentration of archaea was 10^8^ 16S rRNA gene copies·m^−3^ of air. Similarly, DNA sequencing was also applied for pollen detection, for which airborne samples were collected using a DUO SAS Super 360 (VWR) sampler with an air flow rate of 180 L/min [108]. This sampler has two heads for Petri dishes which are filled with sterile petroleum jelly. The characteristic pollens present were *Fagaceae* and *Cupressaceae*. Another research group used a Hirst-type spore trap to evaluate airborne biota (like fungi, pollen) by high-throughput DNA sequencing, considering the 16S rRNA gene. DNA sequencing could detect the biodiversity in between samples [109]. The development of laser-induced fluorescence-assisted bioaerosol detection comprising narrow bands of discharge spectra is one of the molecular origins of bioaerosols [110].

Loop-mediated isothermal amplification (LAMP) is another useful molecular assay method for pathogen detection. It rapidly amplifies the target DNA with high specificity and efficiency under isothermal conditions. Notomi et al. introduced this method in 2000 [111], and in recent years, it has been further developed by being combined with other molecular detection approaches such as multiplex amplification and reverse transcription. Compared to other molecular detection methods, LAMP has numerous significant advantages, including its rapidity, simplicity (only a water bath or heating block is required to maintain the isothermal conditions), compact and portable design, and ability to use pH indicator dyes as DNA probes. Despite these advantages, LAMP is still considered less successful than PCR methods. The major shortcomings of LAMP are its usage of multiple primers and high risk of carryover contamination, which can lead to false-positive results [94,112].

Limulas test is another fruitful biochemical assay used to detect airborne bacterial endotoxins [113]. The basic principle of this test is that when hemocyte lysate comes into contact with a Gram-negative bacterial endotoxin, it forms a gel. This is a good method that can be applied in bioaerosol sampling and detection systems.

### 4.2. Immunological Detection Method

All immunological detection methods are mainly based on the specific interaction between antigen and antibody, whereby a specific antigen binds only with its specific antigen. The sensitivity and specificity of immunological detection methods rely on the binding strength between the specific antibody and antigen. The detection of bioaerosols can be accomplished using antibodies that bind to proteins or polysaccharides on the cell surface of the organisms to bind to antigens. Based on the transduction technology, immunosensors are classified as electrochemical and optical [94]. With the support of a gold nanoparticle (AuNP)-aided surface acoustic wave (SAW), an immunosensor was developed by Toma et al. to detect a specific *D. farinae* allergen [114]. A sandwich assay with the target allergen and biotinylated detection antibody allowed the AuNPs to bind with detection antibodies via the affinity interactions between streptavidin and biotin. The sensor result was three times higher compared to the absence of AuNP with a LOD value of 2.5 ng/mL. Several immunological methods are commercially for the rapid detection and identification of microbes. Radioimmunoassays (RIAs), enzyme-linked immunosorbent assays (ELISAs), and lateral flow immunoassays (LFAs) have recently been used for pathogen detection [115,116].

#### 4.2.1. Radioimmunoassay (RIA)

This technique was first developed for the measurement of peptide hormones; later, it was extended to detect other biological agents. Antibodies are used to detect the amount of antigen present in the biological sample. It is known that radioimmunoassays rely on competitive binding, in which a radioactively labeled antigen competes for a limited number of antibody binding sites with an unlabeled antigen [117]. As the concentration of unlabeled antigen in the sample increases, the radio-labeled antigen–antibody complex levels decrease. A calibration curve for the known concentration of unlabeled antigen provides information about the amount of unknown or unlabeled antigen present in the biological samples. These assay results are more sensitive and more specific. This technique is most useful for the detection of viral antigens, hormones, drugs, mycotoxins, and early-stage cancers [118].

#### 4.2.2. Enzyme-Linked Immunosorbent Assay (ELISA)

The ELISA is one of the most commonly used immunoassay methods for bioaerosol detection. The sandwich ELISA is the most effective form of ELISA, in which the antibodies are coupled with enzymes such as horseradish peroxidase (HRP), beta-galactosidase, and alkaline phosphatases. In this assay, capture antibodies are immobilized onto the walls of microtiter plates, to which the unknown sample or the target microorganism from biological samples is added. Subsequently, the primary antibody is immobilized over the antigen to facilitate binding. In the following step, the enzyme-conjugated secondary antibody (also called detection antibody) is added, and it forms a strong bond with the target antigen, and the unbound antibodies are removed from the titer plates. Then, the complex seems like the antigen is sandwiched in between two antibodies. It can be detected by adding chromogen at the end, which produces a colored end-product that can be analyzed by using spectrophotometric methods [119,120,121].

#### 4.2.3. Lateral Flow Immunoassay

The lateral flow immunoassay (LFA) is better known for its low cost, easy handling, and rapid format. Unlike the ELISA, this technique does not require any special equipment or trained personnel. The LFA is type of immunochromatographic strip which is developed for the on-site detection of pathogens. This strip contains four different sections: the sample pad, conjugate pad, nitrocellulose membrane, and absorbent pad. When the sample is loaded onto the sample pad, it will migrate to other sections by a simple capillary action. At the conjugation pad, sample conjugates bind with the color-labeled antigen or antibody. Then, it will pass through the nitrocellulose line that was immobilized with the antigen or antibody. Finally, depending on the analytes present in the sample, the color particle can bind to the antibody or antigen immobilized at the test line. The whole process takes only 5–10 min after the addition of the sample [122,123].

#### 4.2.4. Improvement of Immunoassays

It has been observed that when an immunoassay is supported with other advanced techniques, it produces exceptional detection/sensing outputs. Kwon et al. applied microfluidics to an immunosensor to detect Influenza A H1N1/2009 present in aerosol samples [124]. The immunosensor could achieve a LOD of 1 and 10 pg/mL when a spectrometer and cell phone camera were used as optical detectors. Similarly, a real-time quartz crystal microbalance (QCM)-based immunosensor has been developed for the detection of the airborne *Felis domesticus* allergen [125]. It was mainly fabricated by the immobilization of antibodies (monoclonal) with a self-assembled cysteamine monolayer. The sensor’s ability to distinguish between dust and the airborne allergen was explored using scanning electron microscopy. Another type of QCM-based piezoelectric immunosensor was reported for *E. coli* identification and quantification in bioaerosols, and this immunosensor was coupled with sampling cyclone SASS 230082 [126]. It took 40 min to complete the process and could show a LOD of 1.45 × 10^4^ CFU/L of air. For airborne *B. anthracis* spores, an immunosensor was developed and coupled with a piezoelectric-excited millimeter-sized cantilever (PEMC) [127]. The immunosensor detection process was near real-time and had a LOD of 5 spores/L. Surface plasmon resonance has also been incorporated into immunosensing applications for the detection of airborne viruses [128]. The recognition of virus presence could be achieved within 2 min, and the total duration for the whole experiment (sampling and analysis) was 6 min. Thus, the addition of advanced tools with immunoassays may lead to significant outcomes for the on-site sampling and detection of bioaerosols. An immunosensor based on a multiplexed SPR-sensor was combined with a personal bioaerosol sampler for the detection of airborne viruses and bacteria with broad concentration ranges in a few minutes [129]. The limitation of the sensor was that it only detected two viral bioaerosols, namely influenza A virus and the MS2 bacteriophage.

Another research group constructed a fiber-optic fluorescent immunoassay system for the quantification of airborne allergens, applying a plastic optical fiber as a photomultiplier sensor probe as part of the detection strategy [130]. The detection level for the sensor was 0.49–250 ng/mL, which resembles that achieved with the enzyme-linked immunosorbent assay (ELISA). An example of a portable immunosensor, known as ‘ImmunoSMART’, was also developed and connected to a cyclone sampling system to detect *E. coli* (DH5α) in bioaerosols [131]. It took 20 min measurement time to provide a LOD of 150 CFU/L of air.

### 4.3. Non-Immunodetection Methods

Apart from PCR and immunoassays, people are working constantly to rapidly and effectively monitor bioaerosols through employing other advantageous techniques. For example, recently, Hyunsoo and coworkers monitored and detected biological molecules in in bioaerosols using UV-LED and light-induced fluorescence measurements [132]. They used a special inkjet aerosol generator to produce an M13 bacteriophage aerosol of fixed size and subsequently monitored it. This inkjet aerosol generator is known for its ability to develop narrow-sized biological particles [133]. Yong-Le et al. developed another advanced optical method to monitor bioaerosols, circular intensity differential scattering (CIDS) [134]. They used a 32-anode photomultiplier tube, along with an elliptical reflector, to record the phase function of scattering. The particle detection ability of the method was around 50,000 particle/second. It was employed to measure aggregates of *Escherichia coli*, *Bacillus subtilis* spores, *Yersinia rohdei*, the bacteriophage MS2, and other molecules, and it was successful in differentiating between single bioaerosol and non-bioaerosol particles. Ultraviolet laser-induced fluorescence (UV-LIF) bioaerosol sensors have become well established in bioaerosol research, but the high cost of these technologies is a prime concern, causing them to be neglected. The improvement of cost effectiveness is a goal aerosol researchers. In 2014, Saari et al. developed a new and economical fluorescence spectrum sensor made up of inexpensive ultraviolet LEDs (280 and 350 nm) and a laser diode (405 nm) [135]. A laser diode-based (405 nm) online bioaerosol detector has also been demonstrated to have potential, with a fluorescent particle fraction (0.34–0.77) in fungal spores and bacteria. A single-particle bioaerosol detector was introduced in the referenced study by Kaliszewski et al., where a continuous wave laser (375 nm) was used on a mobile platform [136]. This device is capable of measuring the fluorescence of single particles even in remote locations online, for which it contains an internal computer and other requirements. This type of technology is very helpful for rapid on-site detection. Wideband Integrated Bioaerosol Sensor (WIBS) 4+ model has been modified to differentiate pollen/fungal spore types utilizing chlorophyll fluorescence [137]. Two additional fluorescence channels have been added to the original sensor to discriminate grass and herb pollens from others. Interestingly, it became successful at solving this issue. 

**Figure 2:**
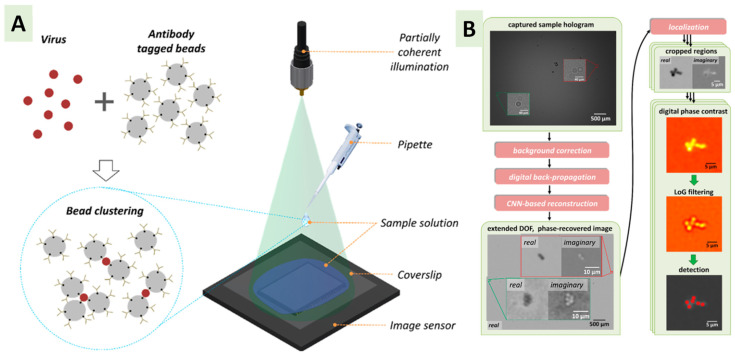
Deep learning-based analysis of particle aggregation-based biosensors. (**A**) Mixing process of solution containing the bioaerosol material with antibody-tagged microparticles, leading to clusters. The solution is put in a plasma-treated coverslip, and a hologram is captured. (**B**) Image processing of clusters. Reprinted with permission from ref [139]. Copyright 2018 American Chemical Society.

A system incorporating single-walled carbon nanotubes (SWCNTs) was demonstrated to act as an electrochemical biosensor for airborne *Bacillus subtilis* [138]. This system operates through transducing the antigen/antibody reaction to produce an electrical signal. The LOD for *B. subtilis* quantification was calculated to be 100 CFU/mL, with a linear range (10^2^–10^10^ CFU/mL). Wu et al. developed particle-aggregation-based biosensors for the quantitative detection of herpes simplex virus (HSV) [139]. A schematic diagram of the virus sensing and imaging is shown below in Figure 2. It requires an antibody specific to HSV, which helps antibody-coated beads to aggregate in the presence of HSV. In the end, the hologram collected is processed to obtain a clear picture of the virus using a deep learning technique. So, it represents a typical sensing/detection platform for bioaerosols.

In some cases, particularly in the culture-dependent methods mentioned above, people use a cultivation system to allow the microbial particles collected from the bioaerosols to grow different colonies and then proceed with analysis and detection [140]. However, this system can only confirm a small fraction (1% of bacteria and 17% of fungi) of the bioaerosol species due to absence of pure cultures [141]. In fact, it was found that in culture-dependent methods, the microbial concentration is 1000 times lower than in PCR or fluorescent microscopy, impacting monitoring and detection [142]. It is noted that most of the previous methods of sampling and detection involve the use of the traditional culture technique, as it is common and well known. However, the limitations observed in its use restrict researchers tremendously, and there is a demand for more sophisticated methods to obtain pure culture and target specific measurements.

**Figure 3:**
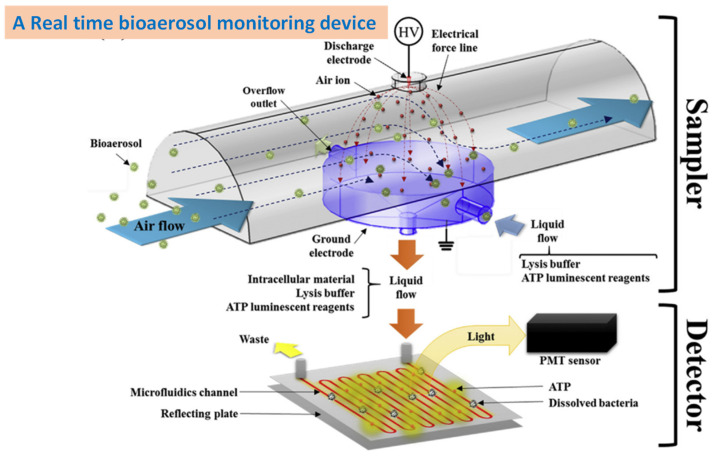
A microfluidics-assisted real-time bioaerosol monitoring device for *S. epidermidis*. Reprinted with permission from ref [143].

Another hybrid system consisting of an aerosol-to-hydrosol sampler and a bioluminescence detector has been developed for real-time airborne *S. epidermidis* sensing. Figure 3 showcases the whole process [143]. The sample is collected under the influence of discharge electrodes in a running liquid with cell lysis buffer and ATP bioluminescence reagents as media and a microfluidic chip and photomultiplier tube (PMT) for detection. The best part of this sensor is that lower concentrations of the bioaerosol can be detected with a lower flow rate of liquid sampling.

## 5. Summary

The field of bioaerosol sampling is both intriguing and challenging, having experienced tremendous growth over the past few decades. While the existing methods have made a significant impact on the bioactivity of bioaerosols, further investigation is necessary to better understand the risk levels associated with these particles. As a result of the greater charge or mass of microorganisms, traditional sampling methods tend to have higher collection efficiency for bioaerosols containing microorganisms on the micrometer scale. However, due to the low concentration of microorganisms in the air, a large number of samples are required to collect enough bioaerosols, which necessitates more efficient collection methods. Moreover, most existing detection methods for pathogens are unable to differentiate between live and dead microorganisms, leading to an overestimation of the pathogen’s presence. Therefore, further research is needed to develop detection methods that can distinguish between live and dead pathogens. There many factors that need to be considered while sampling to obtain maximum bioaerosol quantity, including the sampler’s capacity to hold the materials, the sampling duration, the sampling temperature, the transportation of samples, the selection of an appropriate detection method, the loss of microbes during storage, etc. Also, the sensitivity of the method, LOD, and errors during calculations due to low concentrations and the sampler’s limitations are some other considerations for bioaerosol researchers. Apart from the conventional PCR and immunoassay-based testing, people are now working on the development of new and effective methods to obtain the maximum number of samples and accurately determine the biological materials present. These methods include microfluidics, fluorescence, electrochemical measurements, surface plasmon resonance, piezoelectric behavior, and the inclusion of a PCR enhancer (with nanomaterials). For example, when people use the culture technique to grow a bioaerosol sample after collection, the sample’s concentration may be at a very low level due to many reasons which sometimes cannot even differentiate the materials. This represents a limitation of the technique. In fact, the output through such a technique was reported to have 1000 times lower concentration than PCR or fluorescence microscopy [141,142]. UV-LIF and QCM are important tools that can be coupled with an early detection system to achieve an improved, easy, and sensitive output. These hybrid platforms are capable of showing efficient LOD values. Further, electrochemical biosensors are precise tools for the recognition of bacteria and viruses in bioaerosols samples [94]. A performance comparison between a QCM immunosensor and an electrochemical biosensor designed for the detection of *E. coli* in bioaerosol samples revealed that the LOD of the electrochemical biosensor (150 CFU/L) was lower than the LOD of the QCM immunosensor (1000 CFU/L) [144]. A key area of focus should involve integrating artificial intelligence and big data with detection methods to predict bioaerosol risk levels and suggest effective mitigation strategies. Nowadays, scientists use multiple techniques in a single platform to reduce the sampling and detection time and improve efficacy. The above descriptions refer to such an approach. For the collection of samples, it is better to use samplers which are leakage-free and can separate the bioaerosol from the other airborne materials on site. By leveraging these technologies, we can enhance our ability to detect and manage bioaerosol contamination and safeguard public health.

## Figures and Tables

**Table 1:** Summary and comparison of sampling methods.

Sampling Method	Type	Pros	Cons
Gravity	Passive	Simple and low cost	Low efficiency
Electrostatic attraction	Passive	Low impaction stress Low pressure Compatible with various collection media	Low efficiency with increased flow rates Microbial viability limited on electrical charge and time
Thermal precipitation	Passive	Low pressure High efficiency for smaller particles	Low flow rate Low efficiency for larger particles
Impaction	Active	Simple and low cost Agar plate can be directly used for collection, minimizing post-collection work	Collection efficiency affected by bioaerosol bounce, overlapping, and bioactivity
Cyclone	Active	Low maintenance High efficiency and recovery	Evaporation due to high shear Cyclone size and geometry dependent capacity
Impingement	Active	Liquid medium collection Widely used including commercial ones	Liquid evaporation High shear affect viability
Filtration	Active	Low cost and easy operation Widely used with various materials	Performance deteriorates over bioaerosol loads Post-collection work required

## Data Availability

The relevant data will be available on request.
